# Collaborative Joint Perception and Prediction for Autonomous Driving

**DOI:** 10.3390/s24196263

**Published:** 2024-09-27

**Authors:** Shunli Ren, Siheng Chen, Wenjun Zhang

**Affiliations:** Cooperative Medianet Innovation Center, Shanghai Jiao Tong University, Shanghai 200240, China; renshunli@sjtu.edu.cn (S.R.); zhangwenjun@sjtu.edu.cn (W.Z.)

**Keywords:** collaborative perception, joint perception and prediction, autonomous driving, multi-agent system, spatial–temporal information sharing, information fusion, performance-communication trade-off

## Abstract

Collaboration among road agents, such as connected autonomous vehicles and roadside units, enhances driving performance by enabling the exchange of valuable information. However, existing collaboration methods predominantly focus on perception tasks and rely on single-frame static information sharing, which limits the effective exchange of temporal data and hinders broader applications of collaboration. To address this challenge, we propose CoPnP, a novel collaborative joint perception and prediction system, whose core innovation is to realize multi-frame spatial–temporal information sharing. To achieve effective and communication-efficient information sharing, two novel designs are proposed: (1) a task-oriented spatial–temporal information-refinement model, which filters redundant and noisy multi-frame features into concise representations; (2) a spatial–temporal importance-aware feature-fusion model, which comprehensively fuses features from various agents. The proposed CoPnP expands the benefits of collaboration among road agents to the joint perception and prediction task. The experimental results demonstrate that CoPnP outperforms existing state-of-the-art collaboration methods, achieving a significant performance-communication trade-off and yielding up to 11.51%/10.34% Intersection over union and 12.31%/10.96% video panoptic quality gains over single-agent PnP on the OPV2V/V2XSet datasets.

## 1. Introduction

Autonomous driving is an important technology to improve the efficiency and safety of the transportation system, which has made great progress in recent decades due to the development of sensor technology [1,2] and intelligent algorithms [3,4]. However, the limited capability of a single vehicle restricts its further development. For example, vehicles can not pay enough attention to the region being occluded and at a distance because of the limitation of perception capability, resulting in safety risks. To solve this problem, autonomous driving with the help of collaboration among road agents, e.g., connected and autonomous vehicles (CAVs) and roadside units (RSUs), has arisen and attracted much attention from the research community. With vehicle-to-everything (V2X) communication, CAVs can share messages with neighboring CAVs or RSUs to enhance the capability to achieve good driving performance beyond the limitation of single-vehicle driving, breaking the limitation of single-vehicle.

Collaborative perception [5] is proposed to exchange the processed sensor information among road agents. It helps vehicles achieve perception beyond the line-of-sight and range-of-view, overcoming the occlusion issue and long-range issue of single-agent perception. Shan et al. [6] and Schiegg et al. [7] have demonstrated the effectiveness of the collaboration. To achieve more efficient collaboration, some works [8,9,10] propose efficient collaboration strategies to save communication costs and increase perception accuracy. To make the collaboration more robust, some works [11,12,13] consider collaboration in non-ideal scenarios, such as interruption and delay of V2X communication and pose error of localization among different agents. These works promote the practical application of collaborative perception in the real world.

However, there are two limitations restricting further application of the collaboration. (1) Most research on collaboration among road agents primarily focuses on perception only, without extending the investigation to subsequent autonomous driving processes. This limitation hinders further application of CAV collaboration. Moreover, considering only collaborative perception can lead to cascade error accumulation across sequential modules, posing risks to the performance and safety of autonomous driving systems. (2) Existing collaboration methods are based on single-frame static information sharing and do not account for temporal information sharing. This limitation hinders the completion of tasks requiring temporal information, such as tracking and prediction. When performing such temporal information-required tasks, collaboration must be conducted at each timestep, resulting in redundant communication loads and noise from irrelevant information.

The purpose of this work is to address the above issues, that is, to find an efficient way to share temporal information in a multi-agent collaboration system and expand the benefits of collaboration to more tasks in autonomous driving. To achieve this, we propose CoPnP, a novel collaborative joint perception and prediction system leveraging effective and communication-efficient spatial–temporal information sharing. In the proposed CoPnP system, we design a task-oriented spatial–temporal information-refinement model, which refines the redundant and noisy multi-frame features to comprehensive spatial–temporal features. The refinement model utilizes a spatial–temporal pyramid network and task-oriented explicit supervision to extract the most relevant spatial–temporal features for the PnP task. Additionally, we propose a spatial–temporal importance-aware feature-fusion model that comprehensively fuses the spatial–temporal features from different agents according to the importance of each feature cell.

The proposed CoPnP system has three key novelties:

- It extends the static single-frame information sharing to a multi-frame spatial–temporal information sharing framework. This allows agents to exchange comprehensive spatial–temporal information with minimal communication overhead in a single round of collaboration. Thereby, the collaborative message is enhanced and the system can support tasks that require temporal information.
- The system fully considers the spatial–temporal importance to the PnP task, ensuring that the most critical information is retained during information sharing, which makes the collaboration effective and efficient.
- The system simultaneously outputs the perception and prediction results decoded from a common fused spatial–temporal feature. This approach directly benefits prediction and mitigates the accumulation of cascade error.

**Figure 1:**
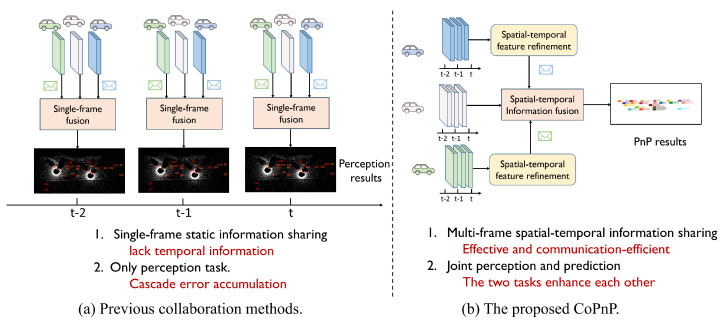
Previous collaboration methods vs. Our proposed CoPnP. Previous methods are based on single–frame static information sharing and only support the perception task. The proposed CoPnP achieves multi-frame spatial–temporal information sharing and benefits more tasks that need temporal information.

Figure 1 compares the proposed CoPnP with previous collaboration methods.

To validate the proposed method for collaborative perception and prediction, we generate PnP labels for two public large-scale collaborative perception datasets, OPV2V [14] and V2XSet [15], and conduct experiments on these two datasets. The OPV2V dataset provides V2V scenarios in diverse traffic scenes, e.g., suburb midblock, urban roads and intersections, and the V2XSet dataset provides vehicle-to-vehicle (V2V) and vehicle-to-infrastructure (V2I) scenarios of 5 different roadway types. The experimental results demonstrate that the proposed CoPnP can outperform existing collaboration methods on the PnP task with a brilliant performance-communication volume trade-off, which can generalize to diverse scenarios and be robust to a certain degree of pose error. CoPnP has a performance gain up to 11.51%/10.34% IoU and 12.31%/10.96% VPQ over single-agent PnP on datasets OPV2V/V2XSet.

The contributions of this paper can be summarized as follows:

(1) We propose CoPnP, a novel collaborative joint perception and Prediction system for autonomous driving, which extends the static single-frame information sharing in previous work to multi-frame spatial–temporal information sharing, expanding the benefits and promoting the application of collaboration among road agents.
(2) To achieve effective and communication-efficient information sharing, we propose two novel designs in the CoPnP system: a task-oriented spatial–temporal information refine model to refine the collaborative messages and a spatial–temporal importance-aware feature-fusion model to comprehensively fuse the spatial–temporal features.
(3) We generate PnP labels for two public large-scale collaborative perception datasets, OPV2V and V2XSet, and conduct experiments on these two datasets to validate the proposed CoPnP. The experimental results show that the proposed CoPnP outperforms existing state-of-the-art collaboration methods on the PnP task with a brilliant performance-communication trade-off and has a performance gain up to 11.51%/10.34% IoU and 12.31%/10.96% VPQ on datasets OPV2V/V2XSet.

The rest of this paper is organized as follows. Section 2 introduces related works. Then, Section 3 formulates the problem and introduces the proposed CoPnP system in detail. Section 4 discusses the experiments and analyzes the results to validate the proposed method. Finally, Section 5 concludes the main contributions of this work and gives some future research directions.

## 2. Related Works

This section introduces the related works, which are divided into two subsections. Section 2.1 introduces existing collaborative perception methods and Section 2.2 introduces the development of joint perception and prediction.

### 2.1. Collaborative Perception

Collaborative perception [5,7,16], which shares perception messages among agents, can overcome the occlusion and long-range issues of individual perception so that agents can achieve good perception performance beyond the line of sight and perception range. Existing methods of collaborative perception can be divided into three basic modes: early collaboration [17], which shares raw observation data, intermediate collaboration [8,15,18], which shares the processed intermediate perception features, and late collaboration, which shares perception results. Some works use mixed collaboration modes, such as DiscoNet [9], leveraging early collaboration as a teacher to guide the learning of a student adopting intermediate collaboration and Arnold et al. [19] conducting late collaboration when the visibility is with high quality and otherwise early collaboration. Both simulated [14,20] and real-world [21,22] datasets are collected to validate the collaborative perception methods.

To promote the application of collaboration perception in real-world scenarios, some works study collaborative perception facing challenging issues. Where2comm [10] proposes to share important features according to spatial confidence for a better trade-off between perception performance and communication volume. SyncNet [12] and CoBEVFlow [23] alleviate the effect of V2X communication delay by estimation from a sequence of features. V2X-INCOP [11] proposes an interruption-aware collaborative perception system that leverages historical information to recover the missing messages. Lu et al. [13] and Vadivelu et al. [24] propose pose-correction methods to make the collaboration perception system robust to pose errors.

Most previous works on collaborative perception only consider single-frame information sharing, which only exchanges static information of one single frame in one round of collaboration, and focuses on the perception task. This hinders collaborative messages carrying more comprehensive spatial–temporal information and prevents the effect of collaboration from benefiting more tasks that need temporal information. In this work, we study how to achieve effective and communication-efficient spatial–temporal information sharing and expand the benefit of collaboration to the PnP task.

### 2.2. Joint Perception and Prediction

Perception and prediction are two important modules of autonomous driving to improve safety [25] and reliability [26,27]. Traditional methods [28,29,30] conduct these two tasks in a cascade manner which first estimates the object detection and tracking results and predicts the object trajectory. This manner depends on the quality of the intermediate results, tends to result in error accumulation, and is unaware of the unknown objects. Some work [31,32] has proposed to conduct perception and prediction in a unified framework for joint reasoning, improving accuracy, robustness, and inference time. Recently, occupancy-based PnP methods [33,34,35], which directly predict semantic occupancy flow to simplify the comprehension of the dynamic scene, have attracted much attention.

Existing works on joint perception and prediction mainly focus on improving single-agent PnP performance and seldom consider the effect of collaboration on PnP performance. In this work, we study how to leverage collaboration among agents to promote the PnP performance on the basis of single-agent PnP.

## 3. Methods: Collaborative Joint Perception and Prediction System

In this section, we introduce the proposed collaborative joint perception and prediction (CoPnP) system. First, we give a problem statement of the addressed task, collaborative joint BEV occupancy perception and prediction. Next, we present the pipeline of CoPnP in detail, which mainly includes: BEV feature extraction, task-oriented spatial–temporal information refinement, message compression, sharing, and decompression, feature transformation, spatial–temporal importance-aware feature fusion, and feature decoding. Finally, we introduce the training loss function of the system. Table 1 provides an explanation of the notations and abbreviations in this paper.

### 3.1. Problem Statement

We first introduce the fundamental definition and goals of the task of collaborative joint BEV occupancy perception and prediction. In a collaboration system, there are some agents located in different regions. To achieve collaboration, they collect observations of their surrounding environment and exchange relevant messages. With the received messages and their own observations, agents need to generate perception results of the current timestep and prediction results for future timesteps.

In this work, we consider that the system takes lidar point clouds collected by different agents at consecutive timesteps as input, including the current timestep and the previous *T* timesteps, and output segmentation maps and occupancy flow in BEV representation for the current perception and prediction for the future $T^{\prime }$ timesteps.

### 3.2. System

**Figure 2:**
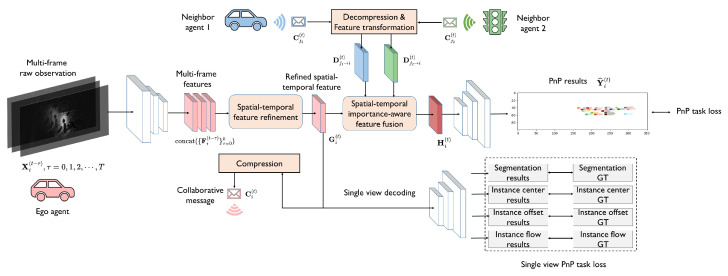
The overall architecture of CoPnP. Given multi-frame extracted features at consecutive timesteps, a spatial–temporal information-refinement model is applied to filter out redundant data and obtain refined spatial–temporal information. Then, a spatial–temporal importance-aware feature-fusion model is used to fuse features from different agents, amplifying important information and suppressing irrelevant information. Finally, the PnP results are generated by a four-head decoder.

System overview. We propose a collaborative joint perception and prediction (CoPnP) system to solve the collaborative joint BEV occupancy perception and prediction task. The overall architecture of the system is shown in Figure 2. In the proposed CoPnP system, each agent collects observations of its surrounding environment at consecutive timesteps, and extracts the features of the raw observation at each timestep. Then we propose a task-oriented spatial–temporal information-refinement model to produce effective and communication-efficient collaborative messages for collaborative PnP. Subsequently, the collaborative messages are compressed by a compression model and then shared among neighbor agents. After message sharing, each agent decompresses the received messages and fuses them comprehensively with a novel spatial–temporal importance-aware feature-fusion model. Finally, we decode the fused features with a four-head decoder to obtain the final perception and prediction results. Next, we will introduce each component in the proposed CoPnP system in detail.

BEV feature extraction. We consider N agents in a collaboration system. Each agent $a_{i}$ collects the raw observations, i.e., the point cloud, of its surrounding environment $X_{i}^{\left(t-\tau \right)}$ at T+1 consecutive timesteps, where τ=0,1,2,⋯,T. When τ=0, it indicates the current timestep, otherwise it indicates the past timestep.

Given the collected point clouds at multiple timesteps from multiple agents, we leverage the backbone of pointpillars [36] as an encoder denoted by $f_{\mathrm{encode}}$ to extract the feature of each point cloud in BEV space, $F_{i}^{\left(t-\tau \right)}$, that is,

$$F_{i}^{\left(t-\tau \right)}=f_{\mathrm{encode}}\left(X_{i}^{\left(t-\tau \right)}\right),\tau =0,1,2,\cdots ,T.$$

Collaborative messages: task-oriented spatial–temporal information refinement. After feature encoding, each agent has a temporal sequence of features. If we directly transmit these features as collaborative messages, it will carry some redundant information. Specifically, the multi-frame features often contain repetitive or non-essential information, due to irrelevant environmental details and temporal correlations across consecutive frames. This redundancy costs many communication resources and introduces noise for collaborative PnP. To produce effective and communication-efficient collaborative messages, the key is to extract the most suitable spatial–temporal features for the PnP task from the multi-frame features and filter out redundant information. To achieve this goal, we propose a task-oriented spatial–temporal information-refinement model.

**Figure 3:**
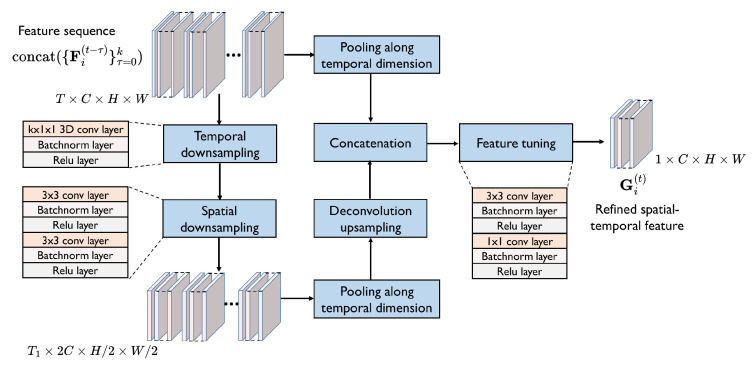
Spatial–temporal information-refinement model. The model refines the feature sequence extracted from multi-frame point clouds to a multi-scale spatial–temporal feature in the following process. The input sequence is first downsampled along both the temporal dimension and spatial dimension. Then the features with two different resolutions are fused by a skip connection. Finally, the refined spatial–temporal feature is obtained by feature tuning.

The main architecture of the refinement model is shown in Figure 3, which extracts multi-scale spatial–temporal features of multi-frame point cloud features. Given the multi-frame features $F_{i}^{\left(t-\tau \right)}$, we concatenate them to a feature sequence as input of the model, that is, concat $\left({\left\{F_{i}^{\left(t-\tau \right)}\right\}}_{\tau =0}^{T}\right)$. We then use a 3D convolution layer with the kernel size of (T,1,1) and two 2D convolution layers to downsample the feature sequence along both the temporal dimension and spatial dimension to obtain spatial–temporal features with two different resolutions. Each convolution layer is followed by a batch normalization layer and a relu function. Subsequently, we conduct a pooling function on the spatial–temporal features along the temporal dimension. Next, we concatenate the higher-resolution features with the upsampled lower-resolution features. That is, a skip connection is applied to fuse the features with different resolutions. Finally, we use two convolution layers to tune the features and generate the multi-scale spatial–temporal features of multi-frame point cloud features, i.e., the refined spatial–temporal feature. The refined spatial–temporal feature of agent $a_{i}$ at the timestep *t*, denoted by $G_{i}^{\left(t\right)}$, is obtained as follows,

$$G_{i}^{\left(t\right)}=f_{\mathrm{refine}}\left(\mathrm{concat}\left({\left\{F_{i}^{\left(t-\tau \right)}\right\}}_{\tau =0}^{k}\right)\right).$$

where $f_{\mathrm{refine}}$ represents the proposed refinement model. As a model extracting temporal features for prediction, the refinement model can effectively capture multi-scale spatial–temporal features. The model inputs all historical information together to avoid cascade errors and has a better prediction for short sequences.

To make the model extract the most suitable features for PnP, we give the refinement model more strong and explicit supervision. Concretely, the multi-scale spatial–temporal features produced by each agent will be not only shared among agents for collaboration but also be decoded to PnP results in the single-agent view supervised by a single-view PnP loss. The ground truth of these results is composed of the semantic segmentation and instance flow, which depicts whether it is foreground and whether it is dynamic at a spatial location and a timestep. The single-view PnP loss can give the refinement model explicit supervision about spatial importance and temporal importance so that the model can realize it and extract the most important information for the PnP task.

The proposed task-oriented spatial–temporal information-refinement model has two advantages: (1) since the single-view feature is also decoded to perception and prediction results and supervised by PnP task loss, the most important information for perception and prediction can be captured and shared among agents; (2) through the refinement model, the size of the collaborative messages to be transmitted is reduced from T×C×H×W to 1×C×H×W, which is communication-efficient because it is not affected by the length of temporal information and is easy to compress.

Thus, $G_{i}^{\left(t\right)}$ carries the most important information for PnP with less communication resources consumption, so is effective and communication-efficient and can serve as the collaborative messages.

Collaborative messages compression, sharing, decompression. After the information refinement, each agent leverages a compression model $f_{\mathrm{compress}}$ to compress the refined features to save more communication resources. Concretely, the compression model uses a 1×1 convolutional layer followed by a batch normalization layer to downsample the channel dimension from *C* to $C^{\prime }$, that is,

$$C_{i}^{\left(t\right)}=f_{\mathrm{compress}}\left(G_{i}^{\left(t\right)}\right).$$

Subsequently, each agent transmits the compressed features $C_{i}^{\left(t\right)}$ as well as its poses in 3D space to its neighbor agents and receives the corresponding messages. Then, each agent decompresses the received messages with a decompression model $f_{\mathrm{decompress}}$, which uses a 1×1 convolutional layer followed by a batch normalization layer to upsample the channel dimension from $C^{\prime }$ to *C*, that is,

$$D_{j}^{\left(t\right)}=f_{\mathrm{decompress}}\left(C_{j}^{\left(t\right)}\right),$$

where $D_{j}^{\left(t\right)}$ is the decompressed feature of collaborative message from agent $a_{j}$ at timestep *t*.

Feature transformation. Next, each agent transforms the features from different agents into its ego coordinate system based on the poses of each agent, that is,

$$D_{j\to i}^{\left(t\right)}=\xi_{j\to i}\left(D_{j}^{\left(t\right)}\right),$$

where $\xi_{j\to i}$ is the transformation principle based on the poses of both agents and $D_{j\to i}^{\left(t\right)}$ is the transformed feature. After this coordinate transformation, all features from neighbor agents $a_{j}$ are aligned within the same coordinate system of agent $a_{i}$.

**Figure 4:**
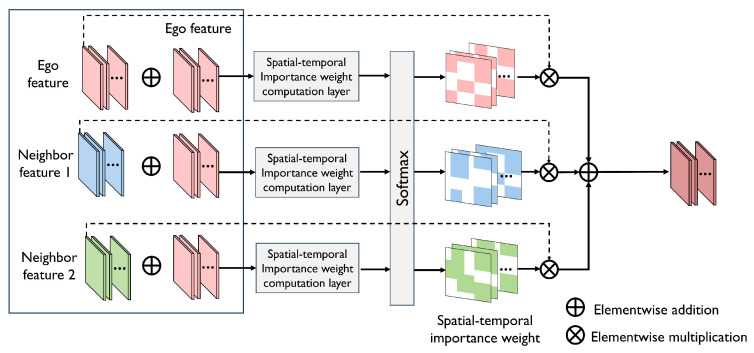
STI-aware feature-fusion model. The model computes the spatial–temporal importance of the feature from different agents to the ego agent, and fuses features according to the computed importance to generate a feature carrying the most important spatial–temporal information for PnP of the ego agent.

Spatial–temporal importance-aware feature fusion. Since different agents have different observations, the importance of the information located at the same spatial and temporal region from different agents is also different. When fusing such information, we should well consider the spatial and temporal importance to amplify the important information and suppress irrelevant information. To achieve comprehensive feature fusion, we propose a spatial–temporal importance (STI)-aware feature-fusion model, which first computes the importance weight of each spatial and temporal cell of the feature and then fuses features based on this weight. See Figure 4.

Given the refined feature from ego agent $G_{i}^{\left(t\right)}$ and the decompressed features $D_{j\to i}^{\left(t\right)}$ from neighbor agents, the STI-aware model first element-wise adds $G_{i}^{\left(t\right)}$ and each $D_{j\to i}^{\left(t\right)}$ and then feeds them into two consecutive convolutional layers to produce a weight feature depicting the spatial–temporal importance of each cell in $D_{j\to i}^{\left(t\right)}$ to $G_{i}^{\left(t\right)}$, whose size is the same as $G_{i}^{\left(t\right)}$ and $D_{j\to i}^{\left(t\right)}$. Following this, a softmax function is applied to normalize the weight features to generate the final weights. The process is denoted as follows:

$$W_{i}^{\left(t\right)}=\mathrm{softmax}\left(f_{\mathrm{weight}}\left(G_{i}^{\left(t\right)}⊕G_{i}^{\left(t\right)}\right)\right),$$

$$W_{j}^{\left(t\right)}=\mathrm{softmax}\left(f_{\mathrm{weight}}\left(G_{i}^{\left(t\right)}⊕D_{j\to i}^{\left(t\right)}\right)\right),j\in N_{i},$$

where $W_{i}^{\left(t\right)}$ is the normalized spatial–temporal weight of agent $a_{i}$’s feature. With the computed spatial–temporal importance weight, we employ weighted averaging to $G_{i}^{\left(t\right)}$ and $D_{j\to i}^{\left(t\right)}$, to achieve the feature fusion considering spatial–temporal importance, that is,

$$H_{i}^{\left(t\right)}=f_{\mathrm{fuse}}\left(G_{i}^{\left(t\right)},D_{j\to i}^{\left(t\right)}\right)=W_{i}^{\left(t\right)}G_{i}^{\left(t\right)}+\sum_{j\in R_{i}^{\left(t\right)}}W_{j}^{\left(t\right)}⊙D_{j\to i}^{\left(t\right)},$$

where $H_{i}^{\left(t\right)}$ is the fused feature. In this way, the STI-aware feature-fusion model simultaneously captures the spatial importance and temporal importance of each cell of the feature maps from different agents so that the most important information to the PnP task can be fused into the final fusion feature.

Feature decoding. Given the fused feature, we apply a multi-head decoder for the PnP task denoted by $f_{\mathrm{decode}}$ to produce the final PnP results. This decoder has four heads that produce the estimation of semantic segmentation, instance centerness, instance offset, and instance flow, respectively. Each head outputs the corresponding results at timesteps from *t* to $t+T^{\prime }$. The semantic segmentation head outputs the results with the size of $(T^{\prime }+1)\times 2\times H\times W$, which is the probability that indicates that each pixel is occupied or not by objects. *H* and *W* are the height and width of the output results, respectively. The instance centerness head outputs the results with a size of $(T^{\prime }+1)\times 1\times H\times W$, which is the probability that indicates that each pixel is an instance center. The instance offset head output results with a size of $(T^{\prime }+1)\times 2\times H\times W$, which is a two-dimensional vector pointing to the instance center. The instance flow head outputs the results with a size of $(T^{\prime }+1)\times 2\times H\times W$, a two-dimensional displacement vector indicating the moving distance of the dynamic objects. The PnP results for agent $a_{i}$ at timestep *t*, ${\hat{Y}}_{i}^{\left(t\right)}$, can be formulated as follows:

$$\begin{array}{cc} {\hat{Y}}_{i}^{\left(t\right)} & ={\left\{\left({\hat{Y}}_{{\mathrm{seg}}_{i}}^{\left(\theta \right)},{\hat{Y}}_{{\mathrm{center}}_{i}}^{\left(\theta \right)},{\hat{Y}}_{{\mathrm{offset}}_{i}}^{\left(\theta \right)},{\hat{Y}}_{{\mathrm{flow}}_{i}}^{\left(\theta \right)}\right)\right\}}_{\theta \in T}\\ \; & =f_{\mathrm{decode}}\left(H_{i}^{\left(t\right)}\right)\\ \; & =\{\left(f_{\mathrm{seg}}\left(H_{i}^{\left(t\right)}\right),f_{\mathrm{center}}\left(H_{i}^{\left(t\right)}\right),f_{\mathrm{offset}}\left(H_{i}^{\left(t\right)}\right),f_{\mathrm{flow}}\left(H_{i}^{\left(t\right)}\right)\right)\}, \end{array}$$

where $T=\{t,t+1,t+2,\cdots ,t+T^{\prime }\}$.

### 3.3. Training Loss

The loss function supervising the training of the proposed CoPnP is composed of a PnP loss $L_{\mathrm{PnP}}$ and a single-view PnP loss $L_{\mathrm{PnP}}\_\mathrm{single}$. The two PnP losses are both composed of four losses supervising the four decoding heads for PnP at the current timestep and the future $T^{\prime }$ timesteps, that is, a segmentation loss $L_{\mathrm{seg}}$, an instance centerness loss $L_{\mathrm{offset}}$, an instance offset loss $L_{\mathrm{offset}}$, and an instance flow loss $L_{\mathrm{flow}}$. The segmentation loss is a pixel-wise cross-entropy loss. The instance centerness loss is an L2 loss and the instance offset loss and the instance flow loss are both L1 losses. The loss of the future timesteps will be exponentially discounted by a parameter β. The total loss $L_{\mathrm{total}}$ can be formulated as follows:

$$\begin{array}{cc} L_{\mathrm{total}} & =L_{\mathrm{PnP}}\left({\hat{Y}}^{\left(t\right)},Y^{\left(t\right)}\right)+\alpha L_{\mathrm{PnP}}\_\mathrm{single}\left({\hat{Y}}_{\mathrm{single}}^{\left(t\right)},Y_{\mathrm{single}}^{\left(t\right)}\right)\\ \; & =\sum_{\theta =t}^{t+T}\beta^{\theta -t}(L_{\mathrm{seg}}\left({\hat{Y}}_{\mathrm{seg}}^{\left(\theta \right)},Y_{\mathrm{seg}}^{\left(\theta \right)}\right)+L_{\mathrm{center}}\left({\hat{Y}}_{\mathrm{center}}^{\left(\theta \right)},Y_{\mathrm{center}}^{\left(\theta \right)}\right)\\ & \;\;\;+L_{\mathrm{offset}}\left({\hat{Y}}_{\mathrm{offset}}^{\left(\theta \right)},Y_{\mathrm{offset}}^{\left(\theta \right)}\right)+L_{\mathrm{flow}}\left({\hat{Y}}_{\mathrm{flow}}^{\left(\theta \right)},Y_{\mathrm{flow}}^{\left(\theta \right)}\right))\\ & \;\;\;+\alpha \sum_{\theta =t}^{t+T}\beta^{\theta -t}(L_{\mathrm{seg}}\_\mathrm{single}\left({\hat{Y}}_{\mathrm{seg}\_\mathrm{single}}^{\left(\theta \right)},Y_{\mathrm{seg}\_\mathrm{single}}^{\left(\theta \right)}\right)+L_{\mathrm{center}}\_\mathrm{single}\left({\hat{Y}}_{\mathrm{center}\_\mathrm{single}}^{\left(\theta \right)},Y_{\mathrm{center}\_\mathrm{single}}^{\left(\theta \right)}\right)\\ & \;\;\;+L_{\mathrm{offset}}\_\mathrm{single}\left({\hat{Y}}_{\mathrm{offset}\_\mathrm{single}}^{\left(\theta \right)},Y_{\mathrm{offset}\_\mathrm{single}}^{\left(\theta \right)}\right)+L_{\mathrm{flow}}\_\mathrm{single}({\hat{Y}}_{\mathrm{flow}\_\mathrm{single}}^{\left(\theta \right)},Y_{\mathrm{flow}\_\mathrm{single}}^{\left(\theta \right)})). \end{array}$$

## 4. Evaluation

In this section, we first introduce the evaluation datasets, metrics, and implementation of the proposed CoPnP. Then, we show the experimental results and corresponding analysis.

### 4.1. Dataset

In this work, we validate our proposed CoPnP system on the task of LiDAR-based joint BEV occupancy perception and prediction. We conduct the experiments on two public collaborative perception datasets, covering V2V, V2I, and V2X scenarios.

OPV2V dataset. OPV2V [14] is a V2V collaborative perception dataset simulated by CARLA and OpenCDA [37]. It includes 11,464 frames of 3D point clouds and 232,913 annotated 3D boxes. Each frame contains 2∼7 vehicles as collaboration agents. Frames totaling 6374/2920/2170 are selected as training/validation/testing samples, respectively. The perception range is x∈[−140 m, 140 m], y∈[−40 m, 40 m ].

V2XSet dataset. V2XSet [15] is a V2X collaborative perception dataset simulated by CARLA and OpenCDA. The scenes of V2XSet cover 5 different roadway types and 8 towns in CARLA and the agent type includes both AVs and infrastructures. It contains 11,447 frames of 3D point clouds split 6694/1920/2833 frames into train/validation/test sets, respectively. The perception range is x∈[−140 m, 140 m], y∈[−40 m, 40 m].

PnP label generation. OPV2V and V2XSet datasets only provide perception labels, i.e., 3D bounding boxes, and no labels for the PnP task. Fortunately, the data are collected and annotated regularly so that we can generate the corresponding PnP labels from the consecutive regularly collected detection annotations following the proposed approach in [33]. We project the 3D bounding boxes into the BEV plane to create an occupancy grid to obtain the segmentation label and then compute the corresponding instance center, offset and future ego-motion.

### 4.2. Metrics and Implementation

Metrics. We use two metrics, intersection over union (IoU) and video panoptic quality (VPQ), for evaluating PnP performance following previous PnP works. IoU is used to measure the quality of segmentation results at each timestep. VPQ is used to measure how consistently the instances are detected over time. The VPQ is defined as:

$$\begin{array}{c} \mathrm{VPQ}=\sum_{t=0}^{H}\frac{\sum_{\left(p_{t},q_{t}\right)\in TP_{t}}\mathrm{IoU}\left(p_{t},q_{t}\right)}{\left|TP_{t}\right|+\frac{1}{2}\left|FP_{t}\right|+\frac{1}{2}\left|FN_{t}\right|}, \end{array}$$

where *H* is the sequence length and $TP_{t}$, $FP_{t}$, and $FN_{t}$ represent the sets of true positives, false positives, and false negatives, respectively.

To validate the communication efficiency, we compute the communication volume of each method by summarizing the float number that needs to be transmitted by an agent during one round of collaboration.

Implementation. We set T=2 and $T^{\prime }=4$, that is, CoPnP takes observations at the past 2 timesteps and the current timestep as input and outputs perception results at the current timestep and prediction results at the future 4 timesteps. To enrich the evaluation scenario, we set the interval between two consecutive timesteps to 0.3 s and 0.5 s, which means the PnP performance of the future 1.2 s and 2 s will be evaluated. For the point cloud feature encoding, we set the width, length, and height of a voxel to be (0.4m,0.4m,4m) and the height, width, and channel of the encoded feature map H=100, W=352 and C=128, respectively. We set the compression ratio of the compression model $C/C^{\prime }=64$. For the training loss, we set the coefficient of the single-view loss α=1, discount coefficient β=0.75, and initial learning rate to be 0.002. We optimize the model using the Adam [38] optimizer. The model is trained on an NVIDIA GeForce RTX A6000 GPU.

### 4.3. Quantitative Evaluation

Baselines and existing methods. As CoPnP is the first work to address the problem of collaborative joint PnP, we compare the proposed CoPnP with some designed baselines and models that integrate existing state-of-the-art collaboration methods with the PnP heads of CoPnP. The baseline methods are introduced as follows.

Single-agent PnP gives PnP results based on the single-agent observation with a Motionnet-like [39] model.

Detection boxes fusion + Kalman filter conducts late fusion of perception results and exchanges the detected box at each past timestep and current timestep, then uses a basic Kalman filter for object tracking and predicting the future state of each object box.

V2VNet [8] takes multi-frame point clouds as input and generates an intermediate feature map, and then aggregates the intermediate features with a graph neural network. Finally, it uses the PnP decoder to generate PnP results.

F-Cooper [18], DiscoNet [9], and Where2comm [10] conduct intermediate feature fusion based on max fusion, a distilled collaboration graph, and feature selection according to spatial confidence, respectively. To compare CoPnP with them on the PnP task, we first use these methods to conduct single-frame collaboration at each timestep and then generate PnP results with the same decoder as our proposed methods.

**Figure 5:**
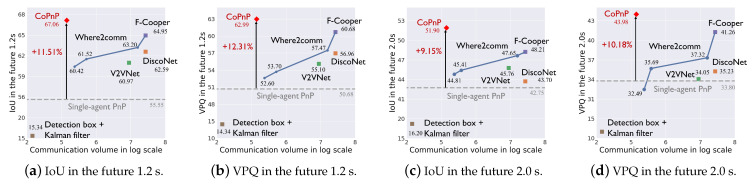
PnP performance with communication volume on the OPV2V dataset.

**Figure 6:**
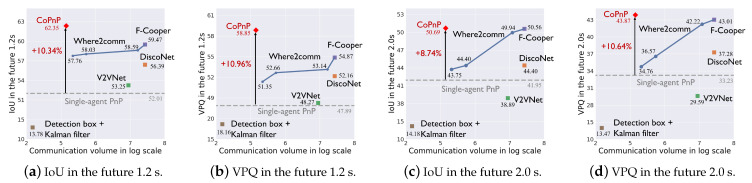
PnP performance with communication volume on the V2XSet dataset.

Benchmark comparison. Figure 5 and Figure 6 show the PnP performance (IoU and VPQ) of each method and corresponding communication volume on datasets OPV2V and V2XSet, respectively. The horizontal axis represents communication volume in the log scale and the vertical axis represents the PnP performance. Each scatter point represents the result of a method and the grey dashed line represents the performance of single-agent PnP. For Where2comm [10], we test its performance with four different communication volumes and with four thresholds of its confidence map, 0,0.01,0.1,0.5.

From the results, we can see the following. (1) Most collaboration methods can outperform the single-agent PnP, reflecting the effectiveness of the collaboration. Detection boxes fusion + Kalman filter fails because the box matching among agents and timesteps raises many errors and generates a lot of false positives. (2) The proposed CoPnP outperforms all the baseline methods in the future 1.2s and 2.0s on the two datasets, achieving 67.06/62.35 IoU, 62.99/58.85 VPQ in the future 1.2s, and 51.90/50.69 IoU, 43.98/43.87 VPQ in the future 2.0 s on the datasets OPV2V/V2XSet. Compared with single-agent PnP achieving 55.55/52.01 IoU, 50.68/47.89 VPQ in the future 1.2 s, and 42.75/41.95 IoU, 33.80/33.23 VPQ in the future 2.0 s on datasets OPV2V/V2XSet, the proposed CoPnP has a gain over single-agent PnP up to 11.51% IoU and 12.31% VPQ on the OPV2V dataset and 10.34% IoU and 10.96% VPQ on the V2XSet dataset. (3) The proposed CoPnP achieves the best performance-communication volume trade-off, whose performance exceeds existing state-of-the-art collaboration methods with 192 times less communication volume. The above results demonstrate that the proposed CoPnP can achieve communication-efficient and high-performance PnP.

Perception only. To validate the perception performance of the proposed method, we test the perception-only results measured by segmentation IoU at the current timestep. Table 2 shows the perception results on the OPV2V and V2XSet datasets. The superscript * indicates that the temporal information (information at the past timesteps) is involved. From the results, we can see that: (1) with temporal information, F-Cooper and DiscoNet can obtain 2.86/3.31 and 5.12/6.46 perception performance gain on datasets OPV2V/V2XSet, respectively, suggesting exchanging temporal information significantly benefits the perception task; (2) CoPnP outperforms the collaborative perception methods based on single-frame collaboration, which makes full use of spatial–temporal information to improve perception performance.

### 4.4. Qualitative Evaluation

**Figure 7:**
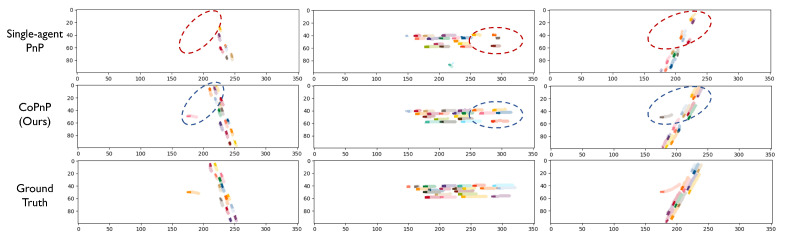
Visualization of PnP results (occupancy flow) on datasets OPV2V and V2XSet. Each column represents an example and the three rows represent the PnP results of single-agent PnP, our proposed CoPnP, and ground truth, respectively. We assign different colors for different vehicles for distinction. The darker color represents the current perception results and the lighter color represents the prediction result in the future. The proposed CoPnP can achieve accurate PnP in the region where single-agent PnP suffers from missing perception and poor prediction performance due to long range and occlusion.

Figure 7 shows the visualization examples of the PnP results of the proposed CoPnP on the datasets OPV2V and V2XSet. Each column represents an example and the three rows represent the PnP results of single-agent PnP, our proposed CoPnP, and ground truth, respectively. We assign different colors for different vehicles for distinction. The darker color represents the current perception results and the lighter color represents the prediction result in the future. From the visualization examples, we can see that single-agent PnP fails in the occluded or long-range region in the red circle, while the proposed CoPnP can achieve accurate PnP results leveraging the collaboration among agents, which validates the effectiveness of our proposed method.

### 4.5. Ablation Study

We conduct an ablation study to validate the effectiveness of the two main designs of CoPnP, the task-oriented spatial–temporal information-refinement model, denoted as STIR, and the STI-aware feature-fusion model denoted as STI-aware fusion. To validate the effectiveness of STIR, we compare it with a vanilla collaboration model based on single-frame collaboration, which conducts single-frame intermediate collaboration with maxfusion and generates results with the Motionnet-like model and PnP decoder mentioned above. To validate the effectiveness of STI-aware fusion, we compare it with maxfusion and discofusion [9].

Table 3 shows the results of the ablation study, which are evaluated by the PnP performance in the future 1.2 s and the communication volume. STIR + maxfusion leverages the proposed STIR model to refine the spatial–temporal features of multi-frame information, and fuses features with maxfusion. STIR + Discofusion replaces the maxfusion with discofusion, which fuses features according to spatial attention. STIR + STI-aware fusion fuses the features with the proposed STI-aware feature-fusion model. The final row shows the result of the proposed CoPnP, which conducts feature compression and decompression with a 64 compression ratio.

From the results, we can see the following. (1) STIR can reduce two-thirds of the communication volume and achieve 65.04/60.16 IoU and 61.20/55.45 VPQ on the datasets OPV2V/V2XSet, outperforming the vanilla model by 0.09/0.69 IoU and 0.52/0.58 VPQ on the datasets OPV2V/V2XSet. This suggests that the STIR model can effectively extract the spatial–temporal features of multi-frame information and filter the redundant information. (2) The STIR + STI-aware feature-fusion model achieves 67.16/62.68 IoU and 63.08/59.14 VPQ on the datasets OPV2V/V2XSet, outperforming maxfusion by 2.12/2.52 IoU and 1.88/3.69 VPQ, and outperforming discofusion by 0.72/1.45 IoU and 0.79/2.09 VPQ on the datasets OPV2V/V2XSet. This demonstrates the effectiveness of STI-aware fusion, which considers both the spatial and temporal importance of each cell of the spatial-temporal feature map, while maxfusion equally considers all cells and the discofusion only considers the spatial importance. (3) The last row shows that the proposed CoPnP exhibited only a slight degradation in performance with a further 64 times compression in communication volume, achieving 67.06/62.35 IoU and 62.99/58.85 VPQ on the datasets OPV2V/V2XSet. This indicates that the information sharing in CoPnP is effective and communication-efficient, which achieves an excellent trade-off between performance and communication volume.

### 4.6. Discussion of the Generalization and Robustness

We discuss the generalization and robustness of the proposed CoPnP based on the experiment results.

Generalization to different scenarios. The OPV2V dataset includes V2V scenarios in diverse traffic and CAV configurations, such as suburb midblock and urban roads. The V2XSet dataset includes V2V and V2I scenarios covering five roadway types, such as entrance ramps and intersections. The results in Figure 5 and Figure 6 show that CoPnP achieves 67.06/62.35 IoU, 62.99/58.85 VPQ in the future 1.2 s, and 51.90/50.69 IoU, 43.98/43.87 VPQ in the future 2.0 s on the datasets OPV2V/V2XSet and outperforms other collaboration methods, suggesting CoPnP can be generalized to diverse scenarios.

**Figure 8:**
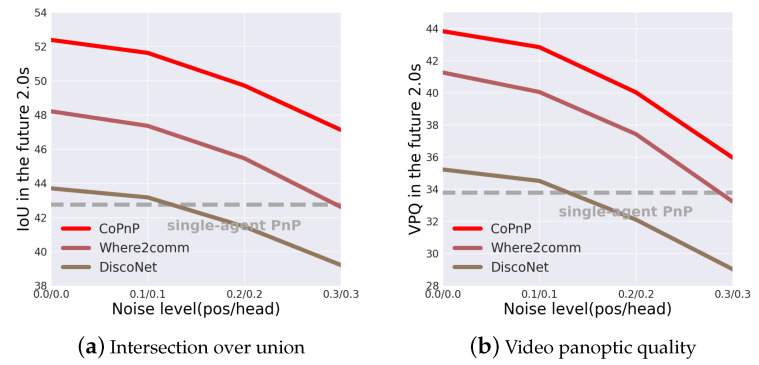
PnP performance with different localization noise on the OPV2V dataset. The horizontal axis represents the localization noise level and the vertical axis represents the IoU and VPQ of the PnP results. The localization noise level is measured by the positional and heading error in Gaussian distribution with zero mean and varying standard deviation.

Robustness to pose error. Correct pose information plays a significant role in fusing information from different agents. However, localization errors are common in the real world. To validate the robustness to pose error of the proposed CoPnP, we test the PnP performance with varying levels of pose error. We use Gaussian noise on the center and yaw angle of the 6DoF pose to simulate the localization error. Figure 8 shows the PnP performance in the future 2.0 s of three collaboration methods with pose error on the OPV2V dataset. From the results, we can see that CoPnP can achieve 52.39/43.83, 51.63/42.84, 49.72/40.03, and 47.12/35.97 IoU/VPQ at pose noise level with 0.0/0.0, 0.1/0.1, 0.2/0.2, 0.3/0.3 standard deviation, respectively, and outperforms the baseline methods. The proposed CoPnP maintains significant gains over single-agent PnP with pose error when the standard deviation of the Gaussian noise is less than 0.3, which demonstrates the proposed CoPnP is robust to a certain degree of pose error.

## 5. Conclusions

This work proposes a collaborative joint perception and prediction (CoPnP) system for autonomous driving, which significantly improves the PnP performance beyond single-agent through efficient spatial–temporal information sharing. Two novel designs are proposed in the CoPnP system, the task-oriented spatial–temporal information-refinement model and the spatial–temporal importance-aware feature-fusion model, which achieve comprehensive spatial–temporal feature refinement and fusion across collaboration agents. Experimental results demonstrate that CoPnP outperforms existing state-of-the-art collaboration methods with a brilliant performance-communication trade-off and has a performance gain up to 11.51%/10.34% IoU and 12.31%/10.96% VPQ over single-agent PnP on datasets OPV2V/V2XSet. This system promotes the application of collaboration among road agents in the real world.

In further research, more autonomous driving modules, such as planning and action controlling, can be considered in a unified framework, which can promote collaboration among road agents directly benefiting the entire process of autonomous driving, resulting in collaborative end-to-end autonomous driving.

## Figures and Tables

**Table 1:** Explanation of notations and the abbreviations for the proper nouns.

Notation	Definition	Abbreviation	Full Name
*T*	Number of past timesteps as input	PnP	Perception and prediction
$T^{\prime }$	Number of future timesteps to predict	CAV	Connected autonomous vehicle
$a_{i}$	Collaboration agent (vehicle or roadside unit)	RSU	Roadside unit
*N*	Number of collaboration agents in the scene	V2X	Vehicle-to-everything
*t*	Timestep	V2V	Vehicle-to-vehicle
$N_{i}$	Set of neighbor collaboration agents of agent $a_{i}$	V2I	Vehicle-to-infrastructure
$X_{i}^{\left(t\right)}$	Raw sensor data observed by agent $a_{i}$ at timestep *t*	BEV	Bird’s eye view
$F_{i}^{\left(t\right)}$	Single-frame features extracted from $X_{i}^{\left(t\right)}$	STI	Spatial–temporal importance
$G_{i}^{\left(t\right)}$	Refined spatial–temporal features of agent $a_{i}$ at timestep *t*	IoU	Intersection over union
$C_{i}^{\left(t\right)}$	Compressed features of $G_{i}^{\left(t\right)}$	VPQ	Video panoptic quality
$D_{i}^{\left(t\right)}$	Decompressed features of $C_{i}^{\left(t\right)}$		
${\hat{Y}}_{i}^{\left(t\right)}$	PnP results of agent $a_{i}$ at timestep *t*		
$Y_{i}^{\left(t\right)}$	Ground truth of ${\hat{Y}}_{i}^{\left(t\right)}$		

**Table 2:** Perception only results. The superscript * indicates the temporal information is involved. The Bold indicates the best results.

	Single-Agent Perception	Late Fusion	V2VNet *	F-Cooper	F-Cooper *	DiscoNet	DiscoNet *	CoPnP *
OPV2V	61.38	73.96	72.21	75.68	78.54	71.33	76.45	**79.43**
V2XSet	56.53	65.05	64.07	66.66	69.97	63.54	70.00	**73.51**

**Table 3:** Ablation study results. STIR represents the proposed task-oriented spatial–temporal information-refinement model. The Bold indicates the best results.

Dataset	STIR	STI-Aware Fusion	OPV2V		V2XSet		Communication Volume in Log Scale
Metric			IoU	VPQ	IoU	VPQ	
Vanilla collaboration model			64.95	60.68	59.47	54.87	7.43
STIR+Maxfusion	✓		65.04	61.20	60.16	55.45	6.95
STIR+Discofusion	✓		66.44	62.29	61.23	57.05	6.95
STIR+STI-aware fusion	✓	✓	**67.16**	**63.08**	**62.68**	**59.14**	6.95
STIR+STI-aware fusion + 64× compression (CoPnP)	✓	✓	67.06	62.99	62.35	58.85	**5.15**

## Data Availability

The OPV2V dataset is available at https://mobility-lab.seas.ucla.edu/opv2v/ (accessed on 24 September 2024). The V2XSet dataset is available at https://github.com/DerrickXuNu/v2x-vit (accessed on 24 September 2024).
